# Salivary Polyamines Help Detect High-Risk Patients with Pancreatic Cancer: A Prospective Validation Study

**DOI:** 10.3390/ijms24032998

**Published:** 2023-02-03

**Authors:** Daisuke Nose, Masahiro Sugimoto, Tsuneo Muta, Shin-Ichiro Miura

**Affiliations:** 1 Department of Cardiology, Fukuoka University Faculty of Medicine, Fukuoka 814-0180, Japan; 2 Department of Cardiology, Fukuoka Heartnet Hospital, Fukuoka 819-0002, Japan; 3 Institute of Medical Science, Tokyo Medical University, Shinjuku, Tokyo 160-8402, Japan; 4 Institute for Advanced Biosciences, Keio University, Tsuruoka, Yamagata 997-0052, Japan; 5 Department of Health Care Center, Imamura Hospital, Saga 841-0061, Japan

**Keywords:** pancreatic cancer, chronic pancreatitis, saliva, metabolomics, polyamine

## Abstract

Pancreatic cancer is one of the most malignant cancer types and has a poor prognosis. It is often diagnosed at an advanced stage because of the absence of typical symptoms. Therefore, it is necessary to establish a screening method for the early detection of pancreatic cancer in high-risk individuals. This is a prospective validation study conducted in a cohort of 1033 Japanese individuals (male, *n* = 467, age = 63.3 ± 11.5 years; female, *n* = 566, age = 64.2 ± 10.6 years) to evaluate the use of salivary polyamines for screening pancreatic diseases and cancers. Patients with pancreatic cancer were not included; however, other pancreatic diseases were treated as positive cases for accuracy verification. Of the 135 individuals with elevated salivary polyamine markers, 66 had pancreatic diseases, such as chronic pancreatitis and pancreatic cysts, and 1 had gallbladder cancer. These results suggest that the salivary polyamine panel is a useful noninvasive pancreatic disease screening tool.

## 1. Introduction

The annual global incidence of pancreatic cancer has doubled in the last 20 years, from 196,000 in 1990 to 441,000 in 2027 [1]. The 5-year survival rate for pancreatic cancer is 11%, the lowest among all cancers [2]. The poor survival is due to diagnosis in the advanced stages in most cases, with only approximately 20% of patients presenting with surgically resectable stages [3]. The five-year survival rates of patients with pancreatic cancer who undergo surgical resection ranges from 15% to 25% [3], and the survival of patients with stage 1A exceeds 80% in the United States [4]. In Japan, a multiple-center screening test for early stage pancreatic cancer was performed and among 200 patients with pancreatic cancer, only 0.7% and 3% were with stage 0 and I diseases, respectively [5]. These studies indicate that the early detection of this cancer is unfavorable.

Several risk factors of pancreatic cancer have been identified. For example, type 2 diabetes increased the risk of developing pancreatic cancer [6]. Current and past smokers were at higher risks than are non-smokers [7], and alcohol consumption (≥30 g per day) increased the risk of developing pancreatic cancer [8]. Particularly, epidemiological studies have reported chronic pancreatitis as a risk factor for pancreatic cancer [9,10,11]. Chronic pancreatitis is a multifactorial fibroinflammatory syndrome which occurs in the pancreas. Repeated inflammation of the pancreas results in extensive fibrotic tissue replacement, leading to chronic pain and exocrine and endocrine pancreatic insufficiency [12]. Considerable variability in the incidence of this disease ranges from 2 to 14 per 100,000 in the United States [13]. Approximately 5% of these patients will develop pancreatic cancer during their lifetime [14]. Compared with healthy individuals, those with chronic pancreatitis have a 13-fold higher risk of developing pancreatic cancer [14]. Thus, the screening of patients with chronic pancreatitis is useful to identify those at a high-risk of developing pancreatic cancer.

Polyamines have been identified as cancer biomarkers [15]. *MYC* regulates the cancer metabolism and promotes glycolysis [16]. *MYC* also promotes aerobic glycolysis in cancer cells and activates glutamate biosynthesis from glutamine, which provides the required energy and substrate. This gene is associated with chemotherapy resistance, intratumoral angiogenesis, epithelial–mesenchymal transition, and metastasis in pancreatic cancers [17]. *MYC* plays a vital role as a master cell proliferation regulator in pancreatic cancer transformation and progression [18]. The overexpression of *MYC* activates the expression of ornithine decarboxylase 1, which encodes a rate-limiting enzyme for polyamine synthesis that catalyzes the production of putrescine from ornithine [19]. Additionally, the activation of spermidine/spermine *N*^1^-acetyltransferase produces acetylated polyamines, which are excreted outside the cell [15,20]. Polyamines are widespread in the surrounding environment and an increase in their concentrations in blood and urine has been observed in various cancers [20,21,22,23]. Polyamine concentrations in the blood and urine are positively correlated with those in pancreatic cancer tissues [24]. Elevated salivary polyamine levels have also been observed in patients with pancreatic cancer and chronic pancreatitis [25]. The detection accuracy for a combination of salivary polyamines using multiple logistic regression (MLR) was compared with tumor markers. For example, the positive detection rates of carcinoembryonic antigen are 0% for chronic pancreatitis and 0, 75, and 47.6% for pancreatic cancers at stage III, IVa, and IVb, respectively. Moreover, those of MLR are 14.3% for chronic pancreatitis and 83.3, 58.3, and 76.2% for pancreatic cancers with Stage III, IVa, and IVb, respectively [25]. Thus, salivary polyamines showed the potential to detect chronic pancreatitis.

The early detection of chronic pancreatitis is still challenging, even though patients show similar clinical phenotypes [26]. The diagnosis of chronic pancreatitis is conducted using computed tomography and magnetic resonance imaging. An endoscopic ultrasound is used to diagnose patients with a high index of suspicion, such as those with recurrent episodes of acute pancreatitis [27]. More cost-effective and low-invasive molecular biomarkers should be identified to establish effective screening methods for chronic pancreatitis [28]. Among various omics technologies, metabolomics-based biomarker discoveries for chronic pancreatitis have also been reported. For example, eight metabolites in the blood were combined, using the Naïve Bayes algorithm, for pancreatic cancer diagnosis [29]. A six-metabolite panel has been identified to diagnose exocrine pancreatic insufficiency [30]. A panel of acetylated polyamines in urine differentiated patients with pancreatic cancer and chronic pancreatitis from healthy individuals [31]. The salivary *N*^1^-acetylspermine levels in patients with chronic pancreatitis are not as high as those in patients with pancreatic cancer, but its level increases in patients with chronic pancreatitis [25]. Thus, polyamines may be used as biofluid markers for the diagnosis of pancreatic diseases, such as pancreatic cancer and chronic pancreatitis.

This study aimed to evaluate the screening potential of salivary polyamines for diagnosing pancreatic diseases. A prospective study was conducted to investigate the relationship between salivary polyamines and each studied pancreatic disease.

## 2. Results

Saliva samples were collected during a health checkup in Miyaki Town, Saga Prefecture, Japan. The inclusion criteria were those who have undergone a physical examination and cancer screening. The exclusion criteria were treatment for any cancer at the time of the study’s initiation, a history of any cancer within 5 years, minors, and pregnant women.

**Figure A1:**
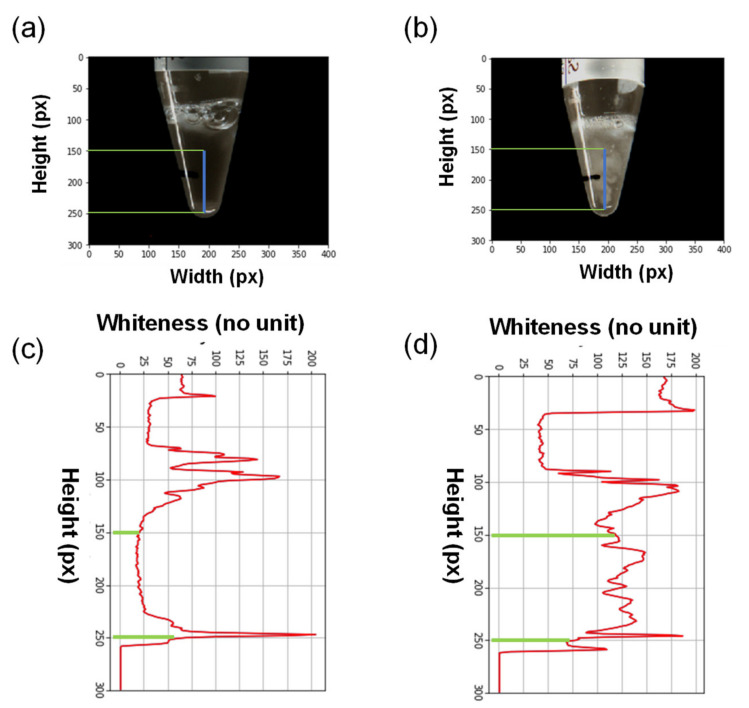
Representative images of acceptable (**a**) and unacceptable (**b**) saliva. The intensity of the whiteness was calculated between the green lines (**c**,**d**). The area between the green lines under the red curves was integrated, and ≥40% of the integrated area between the green lines was considered to represent high whiteness saliva.

The collected saliva samples were immediately stored at −80 °C and transferred to SalivaTech Co., Ltd. (Yamagata, Japan; https://salivatech.co.jp/ (accessed on 1 February 2022)). SalivaChecker (SalivaTech) was used to calculate the risk of pancreatic diseases. This test calculates the cancer risk using an alternative decision tree algorithm using polyamine concentrations in unstimulated saliva samples [32,33]. Overall, higher metabolite concentrations have been observed in stimulated saliva samples than those in unstimulated samples collected from identical individuals [34,35]. As the stimulated saliva turned cloudy and white, the dissolved saliva in a 5 mL tube was photographed in a dark room. The whiteness of the central vertical part was evaluated and the samples with increased whiteness were regarded as unacceptable. The participants were required to secrete saliva again at a later date (Figure A1).

**Figure 1:**
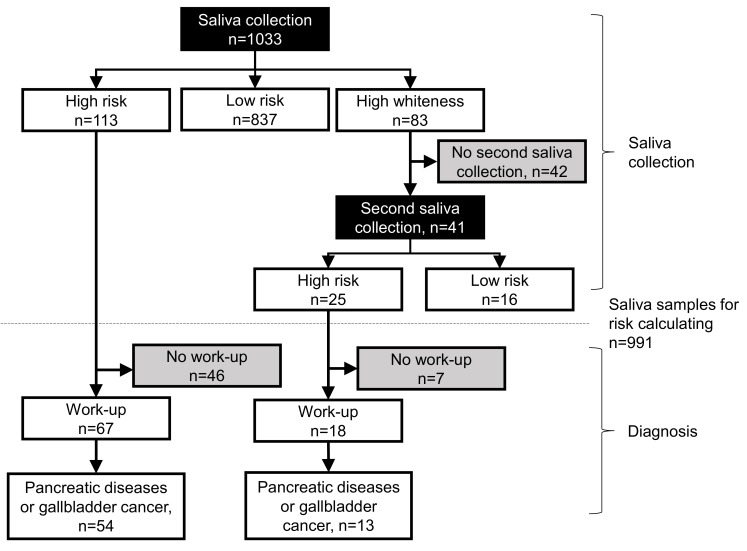
Flowchart of salivary test and diagnosis of pancreatic diseases. Black rectangles indicate saliva tests, and gray rectangles indicate participants that did not undergo saliva tests or pancreatic work-up.

Figure 1 shows the results of the saliva tests. Of the 1033 participants, 113 were identified as having a high-risk and 837 as having a low risk using the saliva tests. Among the high-risk participants, 67 underwent pancreatic work-up, of which 54 were found to have pancreatic diseases, such as chronic pancreatitis. Forty-six participants did not undergo any pancreatic work-up. In addition, 83 participants had high cloudiness in their saliva samples and were subjected to a second saliva collection. Of these, 41 provided saliva, among which 25 participants were at a high-risk, and 16 had a low risk. Among the 25 high-risk participants, 18 underwent pancreatic work-up, and 7 participants did not undergo work-up. Of these 18 participants, pancreatic diseases were observed in 13 participants.

Table 1 shows the characteristics of 991 participants, excluding the ones who did not undergo the second saliva collection (*n* = 42) because their saliva had a high degree of whiteness, which could not be used for calculating the risk. In total, 137 participants showed a high-risk based on the salivary test. Of these 137, 67 patients had pancreatic diseases, including chronic pancreatitis (*n* = 60), pancreatic cysts (*n* = 1), and main pancreatic duct dilatation (*n* = 1). One patient was diagnosed with a gallbladder tumor (*n* = 1), which is not a pancreatic disease but was included as a positive case here.

No significant differences were observed based on the patients’ sex and age (years). The pancreatic disease group included more patients in their 60 s (*n* = 30) and 70 s (*n* = 29) than those ≤ 50 years (*n* = 8). Body mass index was categorized into underweight (<18.5 kg/m^2^), overweight (≥25.0 kg/m^2^), and normal weight between them. The majority of the participants in both the healthy individuals and pancreatic disease groups had a normal weight, with no significant differences. Compared with the odds ratio (OR) for blood pressure in the healthy individual group, that in the pancreatic disease groups was 2.02 (95% CI: 1.18−3.45), which was statistically significant (*p* = 0.009). High-density lipoprotein (HDL) cholesterol was categorized into low HDL (<40 mg/dL) and normal groups (≥40 mg/dL). Low-density lipoprotein cholesterol was categorized into normal (60–120) and abnormal level groups (<60 or ≥120). Both of these data showed no significant differences between healthy individuals and pancreatic disease groups.

Glutamic oxaloacetic transaminase, glutamic pyruvic transaminase, and γ-glutamyl transpeptidase with the thresholds 30, 30, and 50 IU/L, respectively, for the assessment of liver diseases also showed no significant differences in the two groups. Glycated hemoglobin (HbA1c) levels ≥6.5% are used for diabetes diagnosis. Based on this value, no significant differences were observed between healthy individuals and pancreatic disease groups. The triglyceride levels were categorized into two groups, with hypertriglyceridemia (≥150 mg/dL) and normal (<150 mg/dL) groups, which showed no significant differences between healthy individuals and pancreatic disease groups. The parameters for the kidney function (urine sugar, albuminuria, uric blood, estimated glomerular filtration rate, and uric acid levels) showed no significant differences. The current smoking habits also showed no significant differences.

**Figure 2:**
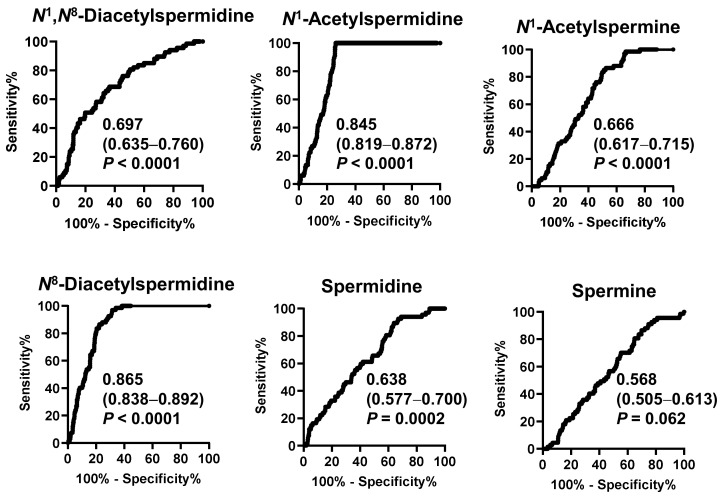
Receiver operating characteristic (ROC) curves to discriminate patients with pancreatic diseases or gallbladder cancer from healthy individuals by salivary polyamines. The area under the ROC curve values, 95% confidential intervals in parenthesis, and *p*-values are shown.

Figure 2 shows the receiver operating characteristic (ROC) curve of salivary polyamines and Table 2 shows the range of the ORs. All salivary polyamines, except spermine, showed significant differences between the pancreatic disease and healthy individual groups. *N*^8^-diacetylspermidine showed the highest area under curve (AUC) value of 0.865 (95% CI: 0.838–0.892), followed by *N*^1^-acetylspermidine (0.845, 95% CI: 0.819–0.872). Their ORs were 1.03 × 10^3^ (95% CI: 78.7–1.5 × 10^4^) and 62.1 (95% CI: 11.4–364), respectively. These factors better indicated a high-risk than blood pressure did.

**Figure A2:**
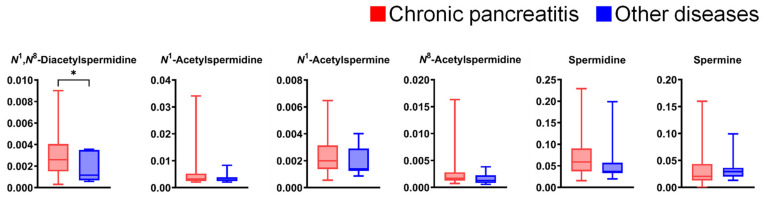
Differences in salivary polyamine concentrations between patients with chronic pancreatitis (*n* = 60) and those with other pancreatic diseases (*n* = 7). Horizontal lines of box plots present 0, 25, 50, 75, and 100% of data. * *p* < 0.05, analyzed using the Mann–Whitney *U* test.

**Figure A3:**
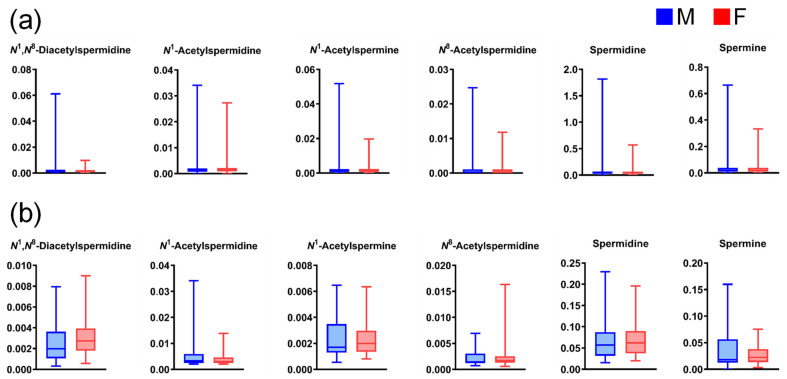
Differences in salivary polyamine concentrations between (**a**) healthy individual male (M) and female (F) participants (*n* = 423 and 501, respectively) and (**b**) M and F patients with pancreatic diseases (*n* = 27 and 40, respectively). Horizontal lines of box plots present 0, 25, 50, 75, and 100% of data. All comparisons show *p* > 0.05, using the Mann–Whitney *U* test.

**Figure A4:**
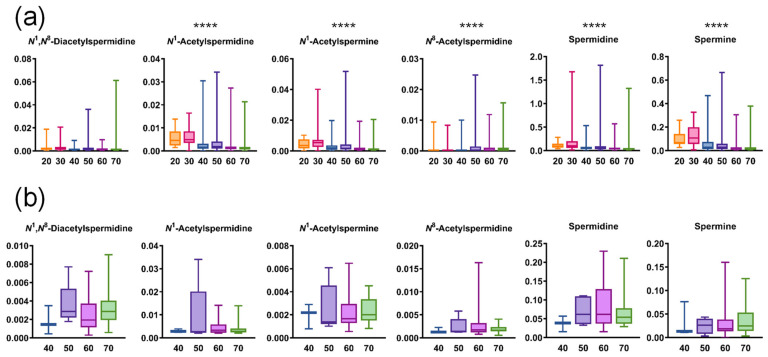
Age-wise differences in salivary polyamine concentrations (**a**) in healthy individuals (20 s, *n* = 17, 30 s, *n* = 23, 40s, *n* = 96, 50 s, *n* = 97, 60 s, *n* = 333, and 70 s, *n* = 358) and (**b**) in patients with pancreatic diseases (40 s, *n* = 3, 50s, *n* = 5, 60 s, *n* = 30, and 70 s, *n* = 20). Horizontal lines of box plots present 0, 25, 50, 75, and 100% of data. **** *p* < 0.0001 using the Kruskal–Wallis test.

**Figure A5:**
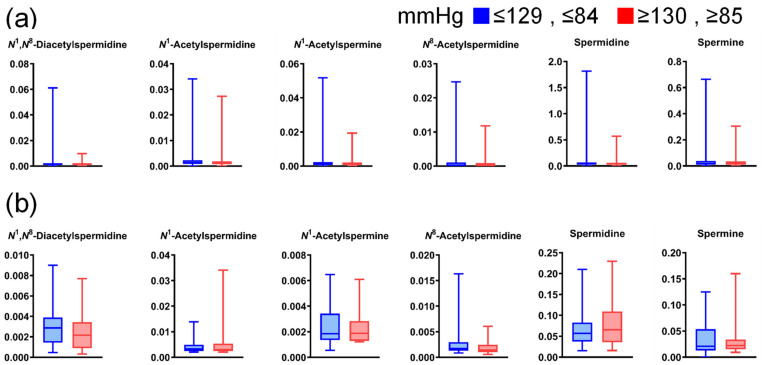
Differences in salivary polyamine concentrations between participants with high and low blood pressure. (**a**) Data for healthy individuals (low, *n* = 744; high, *n* = 180) and (**b**) patients with pancreatic diseases (low, *n* = 45; high, *n* = 22). Horizontal lines of box plots present 0, 25, 50, 75, and 100% of data. All comparisons show *p* > 0.05, using the Mann–Whitney *U* test.

**Figure A6:**
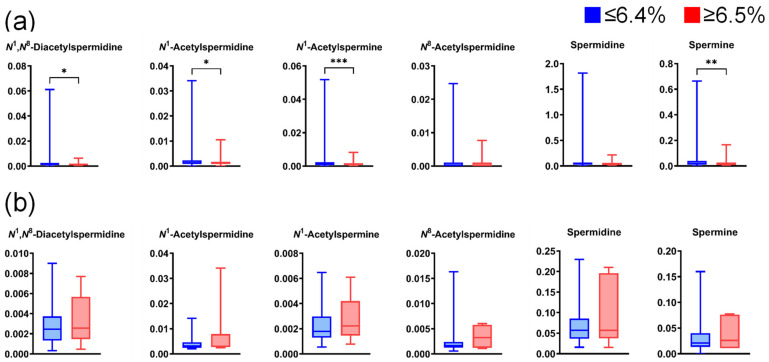
Differences in salivary polyamine concentrations between participants with high and low HbA1c levels. (**a**) Data for healthy individuals (low, *n* = 789; high, *n* = 135) and (**b**) patients with pancreatic diseases (low, *n* = 60; high, *n* = 7). Horizontal lines of box plots present 0, 25, 50, 75, and 100% of data. * *p* < 0.05, ** *p* < 0.01, and *** *p* < 0.001 using the Mann–Whitney *U* test.

Figure A2 presents the comparison of salivary polyamine concentrations between patients with chronic pancreatitis (*n* = 60) and those with other diseases, including pancreatic cysts (*n* = 1) and main pancreatic duct dilatation (*n* = 1). Only *N*^1^,*N*^8^-diacetylspermidine levels were significantly higher in patients with chronic pancreatitis, while the other polyamines showed no significant differences. No polyamines showed significant differences between pancreatic disease and healthy individuals based on sex (Figure A3). Regarding age-specific changes, no statistically significant changes were observed in the pancreatic disease group. In contrast, all polyamines except *N*^1^,*N^8^-*diacetylspermidine showed statistically significant differences in the healthy individual group, which tended to decrease with age (Figure A4). Blood pressure did not affect the polyamine levels in either group (Figure A5). The HbA1c levels showed no significant differences in patients with pancreatic disease. In contrast, the levels of several polyamines were significantly lower in participants with high HbA1c levels in the healthy individuals group (Figure A6). The current smoking habits showed no significant differences in the patients with pancreatic disease and healthy individuals.

## 3. Discussion

This study evaluated the feasibility of screening for patients with a high-risk of pancreatic cancers using salivary polyamines. The potential of salivary metabolites has been reported for oral cancer screening. Salivary metabolites can also detect cancers in organs distant from the oral cavity using saliva [36]. In this study, we evaluated salivary polyamines, the levels of which increased in chronic pancreatitis and pancreatic cancer [25].

The pancreas has higher polyamine levels than that in other organs and is highly sensitive to a polyamine homeostasis disruption [37]. In colorectal cancer, *MYC* mutations occur in adenomas, and drastic changes in downstream metabolic enzymes and the metabolism have been observed [38]. *MYC* mutations have been reported in pancreatic cancer and chronic pancreatitis [39]. These results possibly suggest increased polyamine levels, which is a consequence of *MYC* transcription, in chronic pancreatitis.

In this study, the risk of pancreatic cancer could not be evaluated because pancreatic cancer cases were not included. Therefore, a larger population should be included, considering the low prevalence of pancreatic cancer. This saliva test could detect other pancreatic diseases, including chronic pancreatitis, pancreatic cysts, main pancreatic duct dilatation, and gallbladder tumors. Pancreatic cysts are benign but have a high probability of becoming cancerous during rapid cyst growth [40,41]. The dilatation of the main pancreatic duct is also a potential risk factor for cancer [42]. Our results highlight the possibility of using salivary polyamines for detecting high-risk pancreatic cancer.

It is critical to differentiate between pancreatic cancer and chronic pancreatitis, which can be attempted through blood and urine metabolomic studies. Using machine learning methods, a combination of carbohydrate antigen 19-9 (CA19-9) and nine serum metabolites differentiated pancreatic cancer from chronic pancreatitis in three independent cohorts [43]. Liquid chromatography–mass spectrometry-based plasma metabolomics identified that glycocholic acid, *N*-palmitoyl glutamic acid, and hexanoylcarnitine differentiated pancreatic cancer from chronic pancreatitis [44]. The urinary concentrations of three acetylated polyamines, including acetylputrescine, diacetylspermine, and acetylputrescine, showed a high sensitivity to differentiate pancreatic cancer from chronic pancreatitis [31]. In our polyamine profiles, only diacetylspermine was included, which showed a higher concentration in chronic pancreatitis than that in healthy controls. The inclusion of these acetylated polyamines should be evaluated to increase the sensitivity and specificity. Additionally, this study did not include data on tumor markers, such as CA19-9 and carcinoembryonic antigen. The combination of tumor markers and polyamine also should be evaluated.

The accuracy of the salivary polyamine-based test should be evaluated more rigorously. Only the individuals at a high-risk of using the salivary test underwent the work-up, while those without any symptoms on regular cancer screening did not undergo the work-up, which possibly disregarded the false negative. This study treated the samples showing whiteness as unacceptable, and repeat sampling was required for each participant. Since the overall metabolite concentration in saliva was higher in stimulated saliva, unstimulated saliva without whiteness is necessary for the risk calculation. Without repeating the saliva collection, higher risks are calculated for these samples, which would increase the false positive.

The evaluation of the specificity of salivary polyamines in other diseases is also necessary. Polyamines are potential biomarkers for kidney disease [45]. Fourteen types of elevated polyamines have also been reported in the blood and urine for lung and liver cancers [46]. The blood and urine levels of urea cycle metabolites, including polyamines and their substrates, such as arginine and ornithine, differ between patients with liver cancer and healthy individuals [47]. Elevated salivary polyamine levels have been reported in patients with head and neck cancer [48] and breast cancer [49]. *N*^1^-acetylspermidine and spermidine concentrations are elevated in patients with colorectal and breast cancers, and even with the same polyamine, the pattern of concentration increase can differ depending on the cancer type [32,33]. Therefore, even with the use of salivary polyamines, disease specificity should be improved via the use of concentration patterns for each disease as a panel.

*N*^1^*,N*^12^-diacetylspermine is a urinary biomarker for breast and colorectal cancers [21,23]. However, this metabolite was not detected using capillary electrophoresis-mass spectrometry in the saliva collected from patients with pancreatic cancer [25] and other acetylated forms of polyamines showed higher AUC values than that of *N*^1^*,N*^12^-diacetylspermine [32,33]. Therefore, we did not evaluate this metabolite in this study.

Biofluid-based metabolomic studies distinguish between pancreatic cancer and chronic pancreatitis using metabolites, such as sphingomyelin (d18:2/C17:0), phosphatidylcholine (C18:0/C22:6), isocitrate sphinganine-1-phosphate (d18:0), ceramide (d18:1/C24:0), and sphingomyelin (d17:1/C18:0) [50]. Serum phenylacetylglutamine and chenodeoxyglycocholate help distinguish pancreatic cancer from chronic pancreatitis [44]. Such lipophilic metabolites cannot be separated using the currently used pretreatment methods, which are optimized for hydrophilic metabolites. The plasma metabolite patterns obtained using nucleic magnetic resonance can differentiate among pancreatic cancer, chronic pancreatitis, and healthy participants [51,52]. A further evaluation of the specificity of salivary polyamines in chronic pancreatitis and pancreatic cancers is needed.

A limitation for the use of salivary metabolites is that the salivary metabolomic profile may be affected by factors other than cancer [53]. Therefore, we used strict dietary restrictions before the saliva collection. These restrictions were more severe than the saliva collection protocols used in mRNA or protein marker studies [54,55]. For example, the level of salivary metabolite biomarkers for oral cancers decreases in a short fasting period before the saliva collection [56]. Therefore, verification using relatively less strict restrictions is necessary to facilitate this test.

Smoking and diabetes have been identified as risk factors of chronic pancreatitis [57]. In our data, HbA1c levels, with a cutoff of 6.5% for diagnosing diabetes, showed no significant difference between the pancreatic disease group and healthy individuals. Current smoking habits also showed no significant difference in these groups. Statistically significant differences in the blood pressure levels were observed between the pancreatic disease and the healthy individuals groups. Blood pressure did not affect the salivary polyamine levels in either group. Although the HbA1c levels did not affect the pancreatic disease group, several polyamine levels were significantly lower in the healthy individual group with high HbA1c levels. In the healthy individual group, the levels of most polyamines tended to decrease with age. Among them, the spermidine levels declined with age, which is consistent with the results of a previous study [58]. In the current study, pancreatic diseases were detected at 40 years of age, and high salivary polyamine concentrations in young people were expected. Validation using an increased number of young people is also necessary.

## 4. Materials and Methods

### 4.1. Participants

This study was approved by the Ethics Committees of Fukuoka University (U20-05-029, 19 June 2020) and Tokyo Medical University (T2020-0224, 29 October 2020). Written consent was obtained from all the participants. This study was conducted per the principles of the Declaration of Helsinki. Pancreatic diseases were diagnosed according to the clinical practice guidelines for pancreatic cancer [59] using imaging methods such as endoscopic ultrasonography.

### 4.2. Saliva Collection

Two days before the saliva collection, the participants were prohibited from consuming foods containing high levels of polyamines or their precursor ornithine, such as soybeans, nuts, and supplements. One day before the saliva collection, food other than water was prohibited from 21:00. One hour prior to the sampling, extensive exercise, oral hygiene, mouthwash, toothpicks, and smoking were prohibited. The mouth was lightly rinsed with water immediately before the saliva collection. Unstimulated whole saliva was collected through a 1.1 cm diameter straw into a 5 mL polypropylene tube placed on an ice pack. The saliva samples (0.2 cc) were collected within 15 min and the collection was aborted even though sufficient saliva was not collected. Saliva was collected between 8:00 and 11:00. The saliva was immediately stored at -80 °C until the metabolomic analyses.

### 4.3. Metabolomics Analyses

#### 4.3.1. Saliva Pretreatment

Salivary polyamine quantification was performed using SalivaTech Co., Ltd. (Yamagata, Japan; https://salivatech.co.jp/ (accessed on 1 February 2022)). Measurements were performed by modifying a previously reported method [60]. Frozen saliva samples were thawed for 4 min in warm water (40–42 °C) and photographed at a fixed distance in a darkroom to evaluate the whiteness intensity of the saliva samples. Subsequently, 20 μL of saliva and 60 μL of MeOH + NH_4_OH (1%, *v/v*) containing 2.5 μM of internal standards (Table S1) were mixed. The mixture was centrifuged at 15,780× *g* for 10 min at 4 °C, and 40 μL of the supernatant was separately transferred to a fresh tube. It was mixed with 60 μL Milli-Q water and 30 μL was transferred to a vial and subjected to liquid chromatography−mass spectrometry (LC−MS) analysis.

#### 4.3.2. LC−MS Measurement Conditions

The LC system 1290 Infinity (Agilent Technologies, Santa Clara, CA, USA) comprised an HiP sampler, quaternary pump, and column compartment. A triple-quadrupole mass spectrometer (QQQ-MS) was used as the detector. Chromatographic separation was performed using an ACQUITY BEH C18 column (2.1 mm id × 50 mm, 1.7 μm; Waters, Milford, MA, USA) at 40 °C. The mobile phase comprised solvent A (0.1% formic acid and 1.5 mM heptafluorobutyric acid [HFBA] in water) and solvent B (1.5 mM HFBA in methanol) were delivered at a flow rate of 0.4 mL/min. The gradient elution conditions of pump A were 99.0% at 1.0 min, 99.0% at 2.0 min, 60% at 3.5 min, 5.0% at 4.0 min, and 5.0% at 5 min. The run time was 10 min per sample. An ACQUITY UPLC BEH C18 VanGuard Pre-Column (2.1 mm id × 5 mm, 1.7 μm; Waters) was used for the QQQ-MS analysis.

MS detection was conducted using an Agilent Technologies 6460 triple-quadrupole system. The samples were analyzed in the positive ion mode. The instrument parameters were set as follows: drying gas temperature, 350 °C; drying gas flow, 13 L/min; nebulizer, 55 psig; and Vcap, 3500 V. Specific multiple reaction monitoring transitions, the fragmentor voltage, and collision energy were optimized for each analyzed compound (Table S1). Agilent MassHunter Qualitative Analysis and Quantitative Analysis software, including the MassHunter Optimizer and the Dynamic Multiple Reaction Monitoring Mode software (version B.08.02, Agilent Technologies), were used for data processing.

The mixture, including the ISs and standard compounds, was measured in triplicate before the sample’s measurement to ensure consistent quality. The three metabolites 1,6-diaminohexane, histidine-^13^C_6_, and *N*^1^-acetylspermine-d_3_ were used to evaluate the quality of the measurements. Their retention times, peak heights, and full widths at half maxima were confirmed to be within a certain range (Table S2). We repeatedly performed maintenance, such as replacements, and the qualities of these three metabolites were checked again. In addition, quality control saliva samples were measured at the same time and confirmed to be within a certain range (Table S2).

Among polyamines, *N^1^,N^8^*-diacetylspermidine, *N^1^*-acetylspermidine, *N^8^*-acetylspermidine, *N^1^*-acetylspermine, and spermine were measured as the markers of pancreatic diseases [25]. The concentrations of these metabolites were normalized to the sum of creatinine, histidine, and phosphorylcholine concentrations to cancel the fluctuation in the total density of the saliva samples.

### 4.4. Data and Statistical Analyses

χ^2^ tests were performed for the groups of participants with and without pancreatic diseases. In addition, ORs were calculated. Polyamines in saliva were analyzed using ROC curves and AUC values. Mann–Whitney *U* and Kruskal–Wallis tests were used to evaluate the relationship between the participant characteristics and salivary polyamine concentrations. JMP Pro (ver. 16.0.0; SAS Institute Inc., San Diego, CA, USA) and GraphPad Prism (ver. 9.2.0; SAS Institute Inc.) were used for these analyses.

## 5. Conclusions

This study evaluated the presence of polyamines in saliva as a screening test for pancreatic diseases. However, the risk of pancreatic cancer could not be evaluated. Instead, their potential for detecting other pancreatic diseases was evaluated. The salivary polyamine test has the advantages of being noninvasive, involving less physical burden on each participant, and it is effective for screening high-risk patients with pancreatic cancers.

## Figures and Tables

**Table 1:** Participant characteristics.

Feature		Healthy Individuals	PD ^a^	*p*-Value	Odds Ratio	95% Confidence Interval
Sex ^b^	F	501	40	0.384	0.799	(0.482–1.33)
	M	423	27			
Age	20 s	17	0	0.201		
(years)	30 s	23	0			
	40 s	96	3			
	50 s	97	5			
	60 s	333	30			
	70 s	358	29			
BMI	≤18.5	55	3	0.670		
(kg/m^2^)	18.5–24.9	614	48			
	≥25.0	255	16			
Blood pressure	≤129, ≤84	744	45	0.009 **	2.02	(1.18–2.45)
(mmHg)	≥130, ≥85	180	22			
Triglyceride	<150	745	49	0.14	1.53	(0.870–2.69)
(mg/dL)	≥150	179	18			
HDL cholesterol ^c^	≥40	895	64	0.55	1.45	(0.429–4.88)
(mg/dL)	<40	28	3			
LDL cholesterol ^d^	60–120	413	30	0.99	1.00	(0.605–1.61)
(mg/dL)	≤60 or ≥120	511	37			
GOT ^e^	≤30	824	58	0.510	1.28	(0.615–2.66)
(IU/L)	≥31	100	9			
GPT ^f^	≤30	788	59	0.533	0.786	(0.367–1.68)
(IU/L)	≥31	136	8			
γ-GTP ^g^	≤50	783	58	0.687	0.862	(0.417–1.78)
(IU/L)	≥51	141	9			
HbA1c	<6.5	789	60	0.347	0.682	(0.305–1.52)
(%)	≥6.5	135	7			
Urine sugar	(−)	884	65	0.598	0.680	(0.161–2.88)
	(+)	40	2			
Albuminuria	(−)	838	63	0.359	0.619	(0.220–1.74)
	(+)	86	4			
Uric blood	(−)	852	64	0.322	0.555	(0.170–1.81)
	(+)	72	3			
eGFR ^h^	≥60.0	727	58	0.125	0.573	(0.279–1.18)
(mL/min/1.73 m^2^)	≤59.9	197	9			
Uric acid	2.1–7.0	849	63	0.531	0.719	(0.255–2.03)
(mg/dL)	≤2.0 or ≥7.1	75	4			
Smoking ^i^	No	632	58	0.249	0.580	(0.227–1.48)
	Yes	94	5			
Pancreatic diseases and gallbladder cancer ^j^	CP	0	60			
	Cysts	0	5			
	Others	0	2			
Saliva test ^k^	Low-risk	853	0	<0.0001 ****		
	High-risk	71	67			

^a^ Pancreatic diseases (PD), ^b^ females (F) and male (M), ^c^ high density lipoprotein (HDL), ^d^ low density lipoprotein (LDL), ^e^ glutamic oxaloacetic transaminase (GOT), ^f^ glutamic pyruvic transaminase (GPT), ^g^ γ-glutamyl transpeptidase (γ-GTP), ^h^ estimated glomerular filtration rate (eGFR), ^i^ data for current smoking habit was available only for the participants who underwent cancer screening, ^j^ chronic pancreatitis (CP), pancreatic cysts (Cysts), and others include main pancreatic duct dilatation (*n* = 1). Gallbladder cancer (*n* = 1) is included, ^k^ sensitivity 70% is used as a cutoff between high and low risks, ** *p* < 0.01, and **** *p* < 0.0001.

**Table 2:** Odds ratios (ORs) for salivary polyamines between patients with pancreatic diseases or gallbladder cancer and healthy individuals.

Polyamine	OR	95% Confidence Interval
*N*^1^,*N*^8^-diacetylspermidine	26.4	(0.810–9.78 × 10^3^)
*N*^1^-acetylspermidine	62.1	(11.4–3.64 × 10^2^)
*N*^1^-acetylspermine	3.18	(5.39 × 10^−2^–55.0)
*N*^8^-diacetylspermidine	1.03 × 10^3^	(78.7–1.60 × 10^4)^
Spermidine	3.61	(0.191–68.2)
Spermine	0.502	(8.46 × 10^−3^–9.47)

## Data Availability

The data are not publicly available because of ethical and privacy restrictions.

## Supplementary Materials

The following supporting information can be downloaded at: https://www.mdpi.com/article/10.3390/ijms24032998/s1.
