# Supplementary material for: Salivary Polyamines Help Detect High-Risk Patients with Pancreatic Cancer: A Prospective Validation Study
Source: Int J Mol Sci. 2023 Feb 3;24(3):2998. doi: 10.3390/ijms24032998 (PMC9918012; doi:10.3390/ijms24032998)
Supplement: Supplementary file 1 [file ijms-24-02998-s001.zip › ijms-2183083-supplementary.pdf]

Supplementary Table S1 Measurement conditions

Metabolites	Transition (m/z)	Type[a]	MS1	MS2	Retention time (min)
1,6-Diaminohexane	117.1 -> 100.1	IS	117.1	100.1	2.65
Adenosine-$^{13}C_5$	273.1 -> 136.1	IS	273.1	136.1	2.54
Arginine-$^{13}C6,^{15}N_4$	185.1 -> 75.1	IS	185.1	75.1	2.54
Creathinine-d_3	117.1 -> 47.2	IS	117.1	47.2	0.96
Creatine-d_3	135.1 -> 47.2	IS	135.1	47.2	0.96
Glutamine-$^{13}C_5$	152.1 -> 135.1	IS	152.1	135.1	0.86
Histidine-$^{13}C_6$	162.1 -> 115.0	IS	162.1	115.0	0.85
Hypoxantine-$^{13}C_2,^{15}N$	140.0 -> 113.0	IS	140.0	113.0	0.73
Isoleucine-$^{13}C_6,^{15}N$	139.1 -> 92.1	IS	139.1	92.1	0.72
Leucine-5,5,5-d_3	135.1 -> 89.1	IS	135.1	89.1	0.93
Lysine-$^{13}C_6,^{15}N_2$	155.1 -> 90.1	IS	155.1	90.1	0.46
N^1,N^8-Diacetylspermidine-d_6	236.2 -> 103.1	IS	236.2	103.1	0.46
N^1,N^{12}-Diacetylspermine-d_6	293.3 -> 103.1	IS	293.3	103.1	0.72
N^1-Acetylspermidine-d_6	194.2 -> 106.1	IS	194.2	106.1	0.72
N^1-Acetylspermine-d_3	248.3 -> 103.0	IS	248.3	103.0	0.68
N-Acetyl-D-glucosamine-$^{13}C_2$	223.1 -> 205.1	IS	223.1	205.1	0.68
O-Acetyl_L-carnitine-$^{13}C_2$	206.1 -> 85.1	IS	206.1	85.1	3.05
Phenylalanine-d_5	171.1 -> 125.1	IS	171.1	125.1	3.06
Phosphocholine-d_9	193.1 -> 95.2	IS	193.1	95.2	3.18
Spermidine-d_8	154.2 -> 80.2	IS	154.2	80.2	3.17
Spermine-d_8	211.3 -> 120.1	IS	211.3	120.1	0.79
Trimethylamine N-oxide-d_3	85.1 -> 66.2	IS	85.1	66.2	0.79
Tyrosine-$^{13}C9,^{15}N$	192.1 -> 98.1	IS	192.1	98.1	2.86
Adenosine	268.1 -> 135.9		268.1	135.9	2.83
Arginine	175.1 -> 70.1		175.1	70.1	3.53
Creathinine	114.1 -> 86.1		114.1	86.1	3.52
Creatine	132.1 -> 90.1		132.1	90.1	3.00
gamma-butyrobetain	146.1 -> 87.1		146.1	87.1	2.98
Glutamine	147.1 -> 130.0		147.1	130.0	3.99
Histhidine	156.1 -> 110.0		156.1	110.0	3.99
Hypoxantine	137.0 -> 110.0		137.0	110.0	3.22
Isoleucine	132.1 -> 86.1		132.1	86.1	0.41
Leucine	132.1 -> 86.1		132.1	86.1	0.41
Lysine	147.1 -> 84.1		147.1	84.1	2.14
N^1,N^8-Diacetylspermidine	230.2 -> 100.1		230.2	100.1	2.14
N^1,N^{12}-Diacetylspermine	287.2 -> 100.0		287.2	100.0	3.45
N^1-Acetylspermidine	188.2 -> 100.1		188.2	100.1	3.43
N^1-Acetylspermine	245.2 -> 100.0		245.2	100.0	0.37
N^8-Acetylspermidine	188.2 -> 114.1		188.2	114.1	0.37
N-Acetyl-D-glucosamine	222.1 -> 137.9		222.1	137.9	3.71
O-Acetyl_L-carnitine	204.1 -> 85.1		204.1	85.1	3.71
Phenylalanine	166.1 -> 120.0		166.1	120.0	4.26
Phosphocholine	184.1 -> 86.1		184.1	86.1	4.25
Spermidine	146.2 -> 72.1		146.2	72.1	0.84
Spermine	203.2 -> 112.1		203.2	112.1	0.83
Trimethylamine N-oxide	76.1 -> 58.2		76.1	58.2	2.56
Tyrosine	182.1 -> 91.1		182.1	91.1	2.56

a) IS indicates an internal standard.

Supplementary Table S2 Acceptable ranges of measurement

Metabolite	Item	Range
**Histidine-$^{13}C_6$**	**RT (min)**	0.67－0.82
	**Height**	2719－17427
	**FWHM (min)**	0.035－0.076
**1,6-Diamnohexane**	**RT (min)**	2.48－3.11
	**Height**	2600－10319
	**FWHM (min)**	0.045－0.098
**N1-Acetylspermine-d_3**	**RT (min)**	3.89－4.27
	**Height**	4744－27736
	**FWHM (min)**	0.035－0.088
